# The Kynurenine Pathway in Attention-Deficit/Hyperactivity Disorder: A Systematic Review and Meta-Analysis of Blood Concentrations of Tryptophan and Its Catabolites

**DOI:** 10.3390/jcm13020583

**Published:** 2024-01-19

**Authors:** Daniele Cavaleri, Cristina Crocamo, Pietro Morello, Francesco Bartoli, Giuseppe Carrà

**Affiliations:** 1 Department of Medicine and Surgery, University of Milano-Bicocca, Via Cadore 48, 20900 Monza, Italy; d.cavaleri1@campus.unimib.it (D.C.); cristina.crocamo@unimib.it (C.C.); p.morello2@campus.unimib.it (P.M.); francesco.bartoli@unimib.it (F.B.); 2 Division of Psychiatry, University College London, Maple House 149, London W1T 7BN, UK

**Keywords:** attention-deficit/hyperactivity disorder, tryptophan catabolism, kynurenine pathway, kynurenic acid, peripheral blood, systematic review, meta-analysis

## Abstract

Preliminary evidence shows that the kynurenine pathway (KP) may be altered in attention-deficit/hyperactivity disorder (ADHD). We thus conducted a systematic review and meta-analysis exploring the peripheral blood concentrations of tryptophan catabolites (TRYCATs) in people with ADHD. We searched the main electronic databases up to 7th December 2023. Standardised mean differences (SMDs) with 95% confidence intervals (95%CIs) were used to compare TRYCAT concentrations between participants with ADHD and healthy controls (HCs). We included eight studies. Random-effects meta-analyses found higher kynurenine (SMD = 0.56; 95%CI: 0.04 to 1.08; *p* = 0.033; I^2^ = 90.3%) and lower kynurenic acid (SMD = −0.33; 95%CI: −0.49 to −0.17; *p* < 0.001; I^2^ = 0%) concentrations in people with ADHD compared to HCs. Additional analyses on drug-free children with ADHD showed higher tryptophan (SMD = 0.31; 95%CI: 0.11 to 0.50; *p* = 0.002; I^2^ = 0%) and kynurenine (SMD = 0.74; 95%CI: 0.30 to 1.17; *p* < 0.001; I^2^ = 76.5%), as well as lower kynurenic acid (SMD = −0.37; 95%CI: −0.59 to −0.15; *p* < 0.001; I^2^ = 0%) blood levels, as compared to HCs. Despite some limitations, our work provides preliminary evidence on KP alterations in ADHD that may suggest decreased neuroprotection. Further research is needed to clarify the role of the KP in ADHD.

## 1. Introduction

Attention-deficit/hyperactivity disorder (ADHD) is a neurodevelopmental disorder characterised by inattention, hyperactivity, impulsivity, or a combination of these symptoms [1,2]. ADHD is typically diagnosed in childhood, but a significant number of affected children remain symptomatic in adulthood [3,4]. ADHD shows high rates of psychiatric comorbidities, including depression, bipolar disorder, anxiety, substance use disorders as well as an increased risk of suicide [5,6]. It is well established that the aetiology of ADHD involves complex interactions between inherited and environmental factors, with heritability rates estimated to be around 75% in children and several acknowledged triggers existing during the prenatal and perinatal period [7,8]. Nonetheless, the neurobiological mechanisms underlying ADHD are not yet entirely understood [7]. Several candidate biomarkers have been suggested based on recent advances in biochemical and molecular biology [9]. These include immune-inflammatory alterations such as elevated interleukin-6 and reduced tumour necrosis factor-α [10], although these differences seem more pronounced in children than in adults [10,11]. Nonetheless, evidence on immune-inflammatory markers in ADHD is far from being solid, and there is a lack of evidence supporting reliable biological correlates that can be useful for diagnostic and prognostic purposes [9,10,11,12,13]. Among others, the possible role of peripheral levels of the essential amino acid tryptophan (TRP) and its catabolites (TRYCATs) has been suggested [11,12]. TRYCATs are produced through the kynurenine pathway (KP), the main route for the catabolism of TRP [14,15,16,17]. Indeed, albeit representing the substrate for the synthesis of serotonin and melatonin, more than 90% of TRP is catabolised to kynurenine (KYN) [14,15]. Enzymes indoleamine 2,3-dioxygenase (IDO) in the immune system and the brain, as well as tryptophan dioxygenase (TDO) in the liver, are responsible for the conversion of TRP into KYN, representing the rate-limiting enzymes of the KP [14,15,16,17]. The KYN/TRP ratio thus describes the activity of IDO and TDO and is commonly referred to as the “TRP breakdown index”. KYN is then catabolised into kynurenic acid (KYNA) by kynurenine aminotransferase (KAT) isozymes (KAT-2 in the brain), into anthranilic acid (AA) by kynureninase, and into 3-hydroxykynurenine (3HK) by kynurenine 3-monooxygenase (KMO). 3HK is in turn converted to xanthurenic acid (XA) or 3-hydroxyanthranilic acid (3HAA), with the latter being synthesised also from AA. 3HAA is further catabolised to quinolinic acid (QA), the ultimate substrate for the synthesis of nicotinamide adenine dinucleotide (NAD) [18,19,20]. Many TRYCATs are considered biologically active; for instance, KYNA seems to have an established neuroprotective role, and XA and AA have also been suggested as potentially neuroprotective compounds [14,16,21]. On the other hand, 3HK, 3HAA, and QA are generally considered neurotoxic [22,23].

Based on this biological evidence, the KP has been suggested to be involved in abnormalities that may underlie ADHD, including monoaminergic and glutamatergic neurotransmission as well as immune-inflammatory response [14,17]. In particular, cell-mediated immunity stimulates the KP through the brain infiltration of circulating immune cells, the activation of resident microglia and other non-neuronal cells, and the brain influx of blood-derived, pro-inflammatory cytokines and other immune activators [17]. In children with ADHD, subsyndromal immune-inflammatory imbalances and altered TRYCAT concentrations have been concomitantly found, leading to speculation that they may be predisposing factors to neurodevelopmental abnormalities [11]. The balance between neurotoxic and neuroprotective effects of TRYCATs has led to several different hypotheses concerning their role in the pathophysiology of ADHD [11]. Preclinical, e.g., [24,25], and clinical studies, e.g., [26,27], have investigated alterations in TRYCATs, hypothesising that these may correlate with ADHD. Nonetheless, studies assessing blood levels of TRYCATs in subjects with ADHD have reported inconsistent findings to date, and there is no systematic synthesis of the available evidence from the literature in this field. We thus conducted a systematic review and meta-analysis comparing the peripheral blood concentrations of TRYCATs between people with ADHD and healthy subjects.

## 2. Materials and Methods

This systematic review and meta-analysis was carried out according to the Preferred Reporting Items for Systematic Reviews and Meta-Analyses (PRISMA) 2020 Statement [28].

### 2.1. Eligibility Criteria

We included any observational study investigating blood concentrations of TRYCATs and relevant ratios in ADHD as compared to those in healthy control (HC) groups. We included studies investigating TRYCATs either in children and adolescents or in adults. To improve the consistency and comparability of data, we excluded (i) studies published before the publication of the Diagnostic and Statistical Manual of Mental Disorders, 4th edition (DSM-IV), because of the changes made as compared with the previous version (DSM-III) [29]; (ii) samples investigating ADHD (inattention, hyperactivity) symptoms in healthy subjects; (iii) grey literature, conference abstracts, dissertations, and all publications not undergoing a peer-review process.

When information from the same sample was reported in multiple publications, we used the article providing the most comprehensive data to avoid duplication.

### 2.2. Search Strategy and Inclusion Criteria

Systematic searches of the Embase, Ovid MEDLINE(R), and APA PsycInfo (via ProQuest) databases were performed for articles published from 1994 (year of publication of the DSM-IV) to 7 December 2023. The search phrase used was *(tryptophan OR kynurenine OR kynurenic OR kynurenate OR hydroxykynurenine OR hydroxy-kynurenine OR OH-kynurenine OR anthranilic OR anthranilate OR hydroxyanthranilic OR hydroxy-anthranilic or OH-anthranilic OR hydroxyanthranilate OR hydroxy-anthranilate or OH-anthranilate OR xanthurenic OR xanthurenate OR quinolinic OR quinolinate) AND (ADHD OR attention deficit hyperactivity)* across all fields. No language restrictions were applied. After a preliminary screening based on titles and abstracts, full texts were retrieved to evaluate eligibility. Articles were independently screened and read in full text by two authors, and reasons for exclusion were recorded. Any disagreement was resolved by discussion with the other authors until a consensus was reached.

### 2.3. Data Extraction

Two authors independently extracted study data and blindly cross-checked them for accuracy. A data extraction template was used to collect key information from the eligible studies, including (i) author(s) and year of publication; (ii) country; (iii) type of blood sample; (iv) sample characteristics, including size, mean age, sex distribution, and drug treatment; (v) TRYCATs and ratios as assessed.

### 2.4. Data Analysis

Random-effects meta-analyses were performed for TRYCATs if data were available from at least three studies. Two sets of analyses were performed: (i) an overall analysis including people with ADHD, regardless of age and treatment; (ii) a post hoc analysis including only drug-free children with ADHD, in order to deal with the possible confounding effect of age and pharmacological treatment on TRYCATs. Standardised mean differences (SMDs) (Hedges’ *g*) and their 95% confidence intervals (95%CIs), estimated from means and standard deviations (SDs), were used to compare TRYCAT concentrations between people with ADHD and HCs [30]. If raw data for means and SDs were not reported, they were estimated using conventional transformation methods, when feasible [30,31,32]. Similarly, subgroup data were combined into a single group using standard formulae [30]. The statistical significance level was set at *p* < 0.05 (two-tailed). Forest plots were used to summarise the results. Effect sizes were evaluated according to standard cut-offs for SMDs (0.2: small; 0.5: medium; 0.8: large effect) [33]. Heterogeneity across studies was estimated using the I^2^ statistic, defining low, moderate, and high levels of heterogeneity (I^2^ values around 25%, 50%, and 75%, respectively) [34]. Egger’s test was used to assess the potential publication bias of meta-analyses including at least 10 studies [35].

Analyses were performed using Stata Statistical Software, Release 18 [36]. Forest plots were generated with OpenMeta[Analyst] software [37].

## 3. Results

### 3.1. Study Selection and Characteristics

Our systematic searches generated 524 records (351 from Embase, 101 from Ovid MEDLINE(R), and 72 from APA PsycInfo), including 374 unique articles after deduplication.

**Figure 1:**
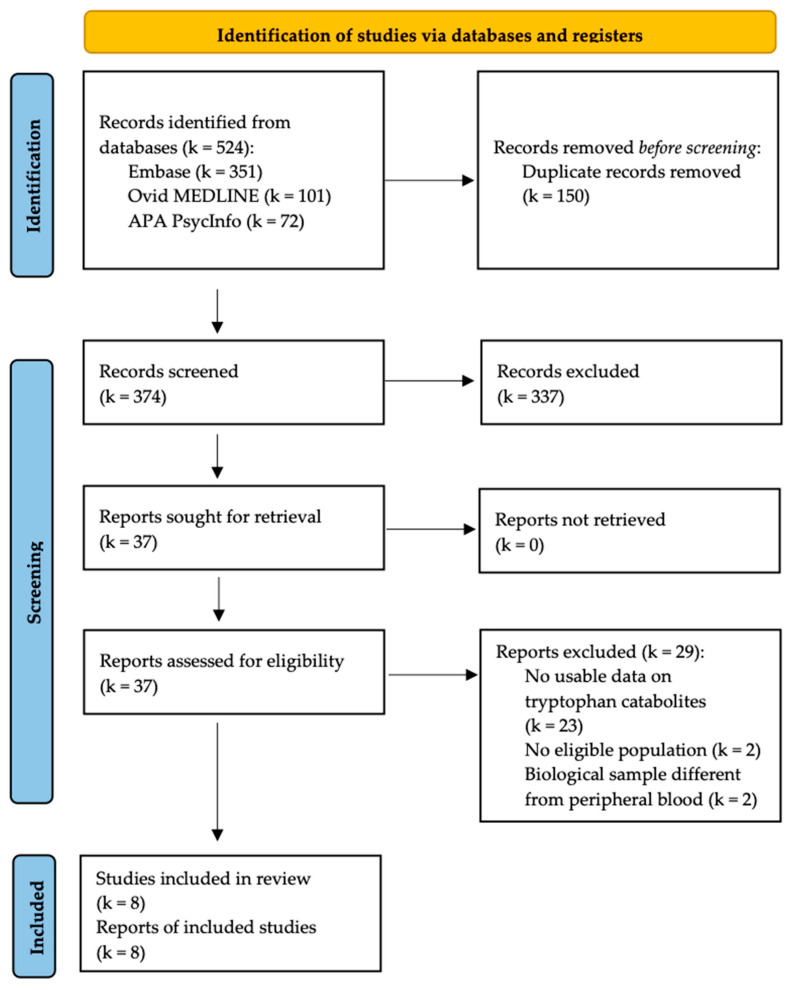
PRISMA flow chart of the inclusion process.

The screening of titles and abstracts identified 37 potentially eligible studies. Among them, eight studies, including a total of 1177 participants (707 with ADHD and 470 HCs) [26,27,38,39,40,41,42,43], met eligibility criteria and were thus included in our meta-analyses. The PRISMA flow chart of the study selection process is reported in Figure 1.

Studies were published between 2010 [43] and 2022 [38]. Sample sizes ranged from 56 [43] to 264 [27]. Consistently with epidemiologic data [44], the male:female ratio in the ADHD sample as a whole was 2.33:1. All studies included children with ADHD, except for the study by Aarsland et al. (2015) which included adults only (18–40 years old) [27]. Six studies tested KP metabolites in serum [26,27,39,40,41,43], one in plasma [38], and one using a dried blood spot technique [42]. Participants with ADHD were all drug-free in five studies [26,38,39,40,41]; of these, two studies [26,39] included drug-naïve subjects only. The study by Oades et al. (2010) provided separate data for drug-naïve (*n* = 21, 60.0%) and medicated (*n* = 14, 40.0%) children with ADHD [43]. The characteristics of the included studies are reported in Table 1.

### 3.2. TRYCAT Concentrations in People with ADHD (Overall Analyses)

**Figure 2:**
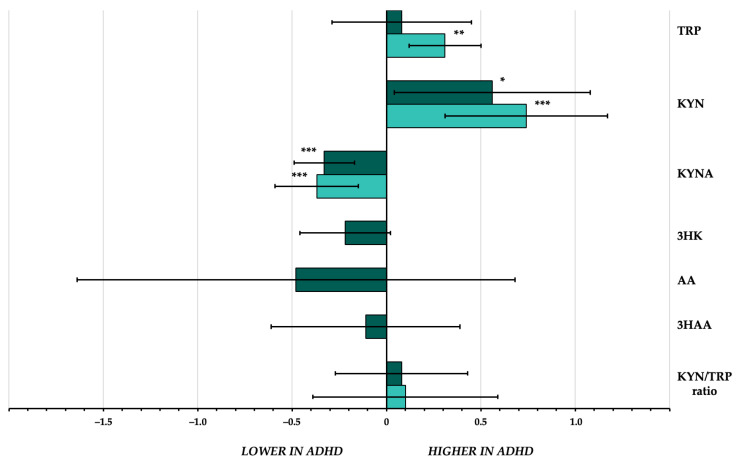
Effect sizes of the differences in peripheral blood concentrations of tryptophan and its catabolites between individuals with attention-deficit/hyperactivity disorder and healthy controls. Darker bars represent effect sizes of overall meta-analyses. Lighter bars represent effect sizes of meta-analyses in drug-free children. Error bars represent 95% confidence interval. * *p* < 0.05; ** *p* < 0.01; *** *p* < 0.001. 3HAA = 3-hydroxyanthranilic acid; 3HK = 3-hydroxykynurenine; AA = anthranilic acid; ADHD = attention-deficit/hyperactivity disorder; KYN = kynurenine; KYNA = kynurenic acid; TRP = tryptophan.

Six components (i.e., TRP, KYN, KYNA, 3HK, AA, and 3HAA) and one ratio (KYN/TRP) of the KP had data available from at least three studies. There were no sufficient data for meta-analyses on other catabolites (XA and QA) and relevant ratios. Overviews of meta-analytic findings are provided in Table 2 and Figure 2.

*Tryptophan*. Six articles, accounting for a total of 909 participants (545 with ADHD and 364 HCs), compared the peripheral blood concentrations of TRP in people with ADHD to those in HCs [26,27,40,41,42,43]. The meta-analysis showed no differences between subjects with ADHD and HCs (SMD = 0.08; 95%CI: −0.29 to 0.45; *p* = 0.68), with high heterogeneity across studies (I^2^ = 85.7%) (Figure S1).

*Kynurenine*. Five studies, including 737 total participants (422 with ADHD and 315 HCs), compared blood KYN concentrations between people with ADHD and healthy subjects [26,27,38,40,43]. The meta-analysis found higher blood KYN concentrations in subjects with ADHD compared to HCs (SMD = 0.56; 95%CI: 0.04 to 1.08; *p* = 0.033), with a medium effect size, though there was high heterogeneity across studies (I^2^ = 90.3%) (Figure S2).

*Kynurenic acid*. Four studies, accounting for 650 participants (389 with ADHD and 261 HCs), compared serum KYNA concentrations in people with ADHD with those of healthy subjects [26,27,40,43]. The relevant meta-analysis showed lower serum KYNA concentrations in people with ADHD (SMD = −0.33; 95%CI: −0.49 to −0.17; *p* < 0.001), with a small-to-medium effect size and no heterogeneity across studies (I^2^ = 0%) (Figure S3).

*3-Hydroxykynurenine.* Serum 3HK concentrations were compared in three studies with 486 participants (287 with ADHD and 199 HCs) [27,40,43]. The meta-analysis showed no differences between subjects with ADHD and HCs (SMD = −0.22; 95%CI: −0.45 to 0.02; *p* = 0.08), with low heterogeneity across studies (I^2^ = 30.4%) (Figure S4).

*Anthranilic acid.* Three studies explored serum AA concentrations in 363 participants with ADHD and 241 HCs [26,27,39]. The meta-analysis did not find differences between the two groups (SMD = −0.48; 95%CI: −1.64 to 0.68; *p* = 0.41), with very high heterogeneity across studies (I^2^ = 97.6%) (Figure S5).

*3-Hydroxyanthranilic acid.* Three studies, including 597 total participants (355 with ADHD and 242 HCs), assessed serum 3HAA concentrations [26,27,40]. No differences between participants with ADHD and HCs were estimated (SMD = −0.11; 95%CI: −0.62 to 0.39; *p* = 0.66), with high heterogeneity across studies (I^2^ = 88.5%) (Figure S6).

*KYN/TRP ratio.* Four studies, including 652 participants (391 with ADHD and 261 HCs), had data on the KYN/TRP ratio (“TRP breakdown index”) in serum [26,27,40,43]. The meta-analysis showed no differences between participants with ADHD and HCs (SMD = 0.08; 95%CI: −0.28 to 0.43; *p* = 0.68; I^2^ = 77.4%) (Figure S7). 

### 3.3. TRYCAT Concentrations in Drug-Free Children with ADHD

Three components (i.e., TRP, KYN, KYNA) and one ratio (KYN/TRP) of the KP had data available from at least three studies testing TRYCATs in drug-free children with ADHD. There were no sufficient data for meta-analyses on other components and relevant ratios. A summary of the meta-analytic findings is provided in Table 3 and Figure 2.

*Tryptophan.* Based on data from four studies [26,40,41,43], we estimated higher TRP concentrations among drug-free children with ADHD (*n* = 315) as compared with HCs (*n* = 162). The effect, though small (SMD = 0.31; 95%CI: 0.11 to 0.50; *p* = 0.002), was consistent (I^2^ = 0%) (Figure S8).

*Kynurenine*. Data available from four studies [26,38,40,43] showed that the concentrations of KYN in drug-free children with ADHD (*n* = 275) were significantly higher than those in HCs (*n* = 185) (SMD = 0.74, 95%CI: 0.30 to 1.17, *p* < 0.001), with a large effect size, though there was high heterogeneity across studies (I^2^ = 76.5%) (Figure S9).

*Kynurenic acid*. Three studies [26,40,43] had data available for the comparison of KYNA levels between drug-free children with ADHD (*n* = 244) and HCs (*n* = 131). The relevant meta-analysis estimated consistent KYNA concentrations with lower levels in ADHD (SMD = −0.37; 95%CI: −0.59 to −0.15, *p* < 0.001; I^2^ = 0%), yielding a small-to-medium effect size (Figure S10).

*KYN/TRP ratio*. According to data from three studies [26,40,43], we did not estimate any difference in the KYN/TRP ratio between ADHD and HCs among drug-free children (SMD = 0.10, 95%CI: −0.39 to 0.59, *p* = 0.68; I^2^ = 77.1%) (Figure S11).

## 4. Discussion

To our knowledge, this is the first work quantitatively synthesising the existing body of evidence on the KP in ADHD. Benefiting from data from eight studies including a total of 1177 participants, several findings regarding the peripheral blood concentrations of TRYCATs in ADHD emerged from our meta-analyses.

First, while the overall meta-analysis on blood concentrations of TRP did not show any variations in ADHD, drug-free children with ADHD showed higher levels than healthy subjects. Although other variables that may influence the peripheral blood levels of TRP (such as diet [45]) were not tested, from these findings, a role of age and medications on blood TRP in ADHD may be hypothesised. Indeed, age is considered one of the main determinants of TRP concentrations and its catabolism [46,47]: systemic TRP levels may physiologically change due to age-related inflammatory activation (i.e., “inflammaging”) [46]. Concerning the possible influence of drug treatment, it has been reported that TRP degradation abnormalities in children with ADHD might be balanced by stimulant treatment [48]. In addition, we found higher blood KYN concentrations in participants with ADHD as compared with HCs in the overall population as well as in drug-free children. Elevated KYN concentrations may result, at least in part, from the increased enzymatic activity of IDO and/or TDO. This mechanism may be driven by the inflammatory stimuli [14,49,50] putatively involved in the pathophysiology of ADHD [10]. However, our meta-analysis on the KYN/TRP ratio did not find any differences between participants with ADHD and HCs. Notably, the substantial heterogeneity observed in both the overall and additional meta-analyses remains unexplained, somewhat reducing the certainty in the evidence on KYN concentrations and KYN/TRP ratio [51]. Therefore, although previous studies have shown that some clinical features of ADHD (such as symptom severity [27] and aggressive behaviours [52]) might be associated with variations in the concentrations of both TRP and KYN, the cumulative available evidence does not allow drawing any firm conclusion regarding the breakdown from TRP to KYN in people with ADHD.

Second, the overall and additional meta-analyses consistently showed that serum KYNA concentrations in people with ADHD were lower than in HCs, with remarkable consistency across studies. A reduction in peripheral KYNA has been previously shown also in other mental disorders [53], such as major depressive [54] and bipolar disorders [55,56], often co-occurring with ADHD [5,57]. Decreased KYNA levels seem to dampen the neuronal activity of midbrain dopamine neurons [58], whose alterations are a shared feature between ADHD [59] and a number of severe mental disorders, including schizophrenia, major depressive disorder, and bipolar disorder [60,61,62]. KYNA is acknowledged to have neuroprotective effects by competitively inhibiting ionotropic glutamate receptors, attenuating activity at the glycine co-agonist site of the NMDA receptor, and antagonising the neurotoxic effects of QA [14,16,63]. Hence, lower KYNA concentrations are consistent with the hypothesis of abnormal glutamatergic transmission in ADHD: reductions in KYNA may limit the endogenous inhibition of NMDA receptors, therefore facilitating receptor activation by the endogenous glutamate in the brains of subjects with ADHD [64,65,66]. The association of lower serum KYNA with higher serum KYN in people with ADHD shown by our meta-analysis may thus corroborate the hypothesis of a decreased activity of enzyme KAT, a key intermediate in the KP which—unlike IDO and KMO—is not induced or upregulated by inflammation [67]. Consistently, although available data were not sufficient to conduct a meta-analysis, some lines of evidence seem to suggest that the ratio of neuroprotective to neurotoxic metabolites KYNA/KYN [19,68] may be reduced in drug-free children with ADHD [40]. Interestingly, it has been shown that serum KYNA concentrations may increase after prolonged treatment with methylphenidate, suggesting a potential neuroprotective effect of treatment with stimulants in ADHD [39].

Finally, no variations of other TRYCATs—namely 3HK, AA, and 3HAA—were found, while no sufficient data were available for other important compounds such as XA and QA. For sure, this calls for further research to investigate the putative neuroprotective effect of XA, whose activity has been linked to brain functioning in ADHD [21,69], especially considering that preliminary data showed lower levels of XA in both children [26] and adults [27] with ADHD. For similar reasons, additional research is needed on the concentrations of QA and its possible neurotoxic effects, also in view of the non-significant findings concerning its direct precursors 3HK and 3HAA. This may be useful also to clarify the role that the hydroxylation of KYN into 3HK (the direct precursor of 3HAA) by KMO might play in ADHD.

Since the available evidence is limited, at present, the peripheral blood levels of TRYCATs cannot be recommended as diagnostic or prognostic biomarkers of ADHD in clinical practice. Additional studies with larger samples are needed to confirm their reliability. Nonetheless, the KP may represent a potential therapeutic target in ADHD. Several approaches to modulating activity along the KP that may have therapeutic potential in the treatment of different neuropsychiatric disorders have been proposed [17,70]. In this context, pharmacological development has focused mostly on the inhibition of enzymes of the KP [17,70]. Specific attention has been paid to inhibitors of KMO: indeed, KMO blockage seems to limit the catabolism through the neurotoxic branch of the KP and subsequently raise KYNA levels, ultimately leading to increased neuroprotection [17,71,72]. In this light, discovering effective KP modulators may also help shed more light on the role of the pathway in ADHD as well as other neuropsychiatric conditions associated with neuroinflammation [71].

Some limitations should be acknowledged when interpreting the findings of our meta-analyses. First, the cross-sectional nature of the included studies does not allow any causal inference about the relationship between TRYCATs and ADHD. Second, although most of the included studies were sufficiently consistent in terms of inclusion criteria and methods to assess TRYCATs, some meta-analyses were characterised by high, unexplained heterogeneity that could not be addressed through subgroup or sensitivity analyses due to the small number of included studies [73]. Finally, most of our analyses were affected by low statistical power because of the limited number of studies and participants included. This also precluded the assessment of both possible moderators (through meta-regression analyses) and publication bias.

## 5. Conclusions

Despite the limited number of included studies and the inconsistency of some findings, our systematic review and meta-analysis provides preliminary evidence on alterations in the KP in ADHD. People with ADHD have higher KYN and lower KYNA blood concentrations than HCs, which may imply decreased neuroprotection. This makes sense for those experimental therapeutic approaches that target the KP by aiming at reducing the production of neurotoxic catabolites in favour of neuroprotective ones such as KYNA [71,72]. Moreover, drug-free children with ADHD are likely to have higher peripheral TRP levels than HCs, but this finding might have been influenced by several confounding factors that could not be addressed and needs further confirmation. However, the available evidence does not allow suggesting the use of TRYCATs as diagnostic or prognostic biomarkers of ADHD in clinical practice yet. Our work warrants additional research to bridge the knowledge gap in this field, aimed at clarifying the role of neuroprotective and neurotoxic KP compounds in ADHD. Future studies should investigate larger cohorts as well as the possible influence of possible moderators such as age, sex, ADHD symptom severity, and treatment.

## Figures and Tables

**Table 1:** Characteristics of the included studies.

Study	Country	Sample	Participants with ADHD				Healthy Controls			Compounds	Ratios
			Sample Size	Age (Years) *Mean ± SD*	Sex *Male % (M/F)*	Drug-Free *%*	Sample Size	Age (Years) *Mean ± SD*	Sex *Male % (M/F)*		
Aarsland et al., 2015 [27]	Norway	serum	133	28.0 ± 9.7	46.6%	31.6%	131	22.5 ± 4.2	42.1%	TRP, KYN, KYNA, 3HK, XA, AA, 3HAA, QA	KYN/TRP
					(62 M/71 F)				(56 M/75 F)		
Bergwerff et al., 2016 [42]	The Netherlands	dried blood spots	83	9.7 ± 1.7	74.7%	39.8%	72	9.9 ± 1.7	51.4%	TRP	–
					(62 M/21 F)				(37 M/35 F)		
Evangelisti et al., 2017 [26]	Italy	serum	102	9.3 ± 2.7	73.5%	100% ^§^	62	9.6 ± 1.7	77.4%	TRP, KYN, KYNA, XA, AA, 3HAA, QA	KYN/TRP
					(75 M/27 F)				(48 M/14 F)		
Kilany et al., 2022 [38]	Egypt	plasma	31	8.2 ± 1.4	64.5%	100%	54	8.5 ± 1.8	64.8%	KYN	–
					(20 M/11 F)				(35 M/19 F)		
Molina-Caraballo et al., 2021 [39]	Spain	serum	130	9.5 ± 2.5	78.5%	100% ^§^	49	10.4 ± 2.6	67.3%	AA	–
					(102 M/28 F)				(33 M/16 F)		
Oades et al., 2010a [43]	Germany	serum	35	10.4 ± 2.5	74.3%	60.0% ^§^	21	11.0 ± 1.5	95.2%	TRP, KYN, KYNA, 3HK	KYN/TRP, KYNA/KYN, KYNA/3HK
					(26 M/9 F)				(20 M/1 F)		
Sağlam et al., 2021 [40]	Turkey	serum	122	11.1 ± 2.5	77.0%	100%	50	11.3 ± 2.7	70.0%	TRP, KYN, KYNA, 3HK, 3HAA	KYN/TRP, KYNA/KYN, KYNA/3HK, 3HK/KYN
					(94 M/28 F)				(35 M/15 F)		
Skalny et al., 2021 [41]	Russia	serum	71	8.4 ± 2.6	76.1%	100%	31	8.0 ± 2.9	77.4%	TRP	–
					(54 M/17 F)				(24 M/7 F)		

3HAA = 3-hydroxyanthranilic acid; 3HK = 3-hydroxykynurenine; AA = anthranilic acid; KYN = kynurenine; KYNA = kynurenic acid; QA = quinolinic acid; TRP = tryptophan; XA = xanthurenic acid. F = females; M = males; SD = standard deviation. ^§^ All drug-naive.

**Table 2:** Concentrations of tryptophan and its catabolites in people with ADHD: summary of meta-analytic findings.

	k	*n*	*n* ADHD	*n* HCs	SMD	95%CI	*p*-Value	*I* ^2^
TRP	6	909	545	364	0.08	−0.29 to 0.45	0.68	85.7%
KYN	5	737	422	315	0.56	0.04 to 1.08	0.033	90.3%
KYNA	4	650	389	261	−0.33	−0.49 to −0.17	<0.001	0%
3HK	3	486	287	199	−0.22	−0.45 to 0.02	0.08	30.4%
AA	3	604	363	241	−0.48	−1.64 to 0.68	0.41	97.6%
3HAA	3	597	355	242	−0.11	−0.62 to 0.39	0.66	88.5%
KYN/TRP ratio	4	652	391	261	0.08	−0.28 to 0.43	0.68	77.4%

3HAA = 3-hydroxyanthranilic acid; 3HK = 3-hydroxykynurenine; AA = anthranilic acid; KYN = kynurenine; KYNA = kynurenic acid; TRP = tryptophan. 95%CI = 95% confidence interval; ADHD = attention-deficit/hyperactivity disorder; HCs = healthy controls; SMD = standardised mean difference.

**Table 3:** Concentrations of tryptophan and its catabolites in drug-free children with ADHD: summary of meta-analytic findings.

	k	*n*	*n* ADHD	*n* HCs	SMD	95%CI	*p*-Value	*I^2^*
TRP	4	477	315	162	0.31	0.11 to 0.50	0.002	0%
KYN	4	460	275	185	0.74	0.30 to 1.17	<0.001	76.5%
KYNA	3	375	244	131	−0.37	−0.59 to −0.15	<0.001	0%
KYN/TRP ratio	3	375	244	131	0.10	−0.39 to 0.59	0.68	77.1%

KYN = kynurenine; KYNA = kynurenic acid; TRP = tryptophan. 95%CI = 95% confidence interval; ADHD = attention-deficit/hyperactivity disorder; HCs = healthy controls; SMD = standardised mean difference.

## Data Availability

Data available from the authors upon reasonable request.

## Supplementary Materials

The following supporting information can be downloaded at: https://www.mdpi.com/article/10.3390/jcm13020583/s1, Figure S1: Blood tryptophan (TRP) concentrations in people with ADHD as compared with healthy controls. Figure S2: Serum kynurenine (KYN) concentrations in people with ADHD as compared with healthy controls. Figure S3: Serum kynurenic acid (KYNA) concentrations in people with ADHD as compared with healthy controls. Figure S4: Serum 3-hydroxykynurenine concentrations in people with ADHD as compared with healthy controls. Figure S5: Serum anthranilic acid (AA) concentrations in people with ADHD as compared with healthy controls. Figure S6: Serum 3-hydroxyanthranilic acid (3HAA) concentrations in people with ADHD as compared with healthy controls. Figure S7: Serum kynurenine/tryptophan (KYN/TRP) ratio in people with ADHD as compared with healthy controls. Figure S8: Blood tryptophan (TRP) concentrations in drug-free children with ADHD as compared with healthy controls. Figure S9: Serum kynurenine (KYN) concentrations in drug-free children with ADHD as compared with healthy controls. Figure S10: Serum kynurenic acid (KYNA) concentrations in drug-free children with ADHD as compared with healthy controls. Figure S11: Serum kynurenine/tryptophan (KYN/TRP) ratio in drug-free children with ADHD as compared with healthy controls. References [26,27,38,39,40,41,42,43] are cited in the Supplementary Materials.
