# Supplementary material for: The Kynurenine Pathway in Attention-Deficit/Hyperactivity Disorder: A Systematic Review and Meta-Analysis of Blood Concentrations of Tryptophan and Its Catabolites
Source: J Clin Med. 2024 Jan 19;13(2):583. doi: 10.3390/jcm13020583 (PMC10815986; doi:10.3390/jcm13020583)

Supplementary Figures

Blood concentrations of tryptophan and its catabolites in people with ADHD (overall analyses).....1

Figure S1. Blood tryptophan (TRP) concentrations in people with ADHD as compared with healthy controls. 1

Figure S2. Serum kynurenine (KYN) concentrations in people with ADHD as compared with healthy controls. 2

Figure S3. Serum kynurenic acid (KYNA) concentrations in people with ADHD as compared with healthy controls. 3

Figure S4. Serum 3-hydroxykynurenine concentrations in people with ADHD as compared with healthy controls. 4

Figure S5. Serum anthranilic acid (AA) concentrations in people with ADHD as compared with healthy controls. 5

Figure S6. Serum 3-hydroxyanthranilic acid (3HAA) concentrations in people with ADHD as compared with healthy controls. 6

Figure S7. Serum kynurenine/tryptophan (KYN/TRP) ratio in people with ADHD as compared with healthy controls. 7

Blood concentrations of tryptophan and its catabolites in drug-free children with ADHD.....8

Figure S8. Blood tryptophan (TRP) concentrations in drug-free children with ADHD as compared with healthy controls. 8

Figure S9. Serum kynurenine (KYN) concentrations in drug-free children with ADHD as compared with healthy controls. 9

Figure S10. Serum kynurenic acid (KYNA) concentrations in drug-free children with ADHD as compared with healthy controls. 10

Figure S11. Serum kynurenine/tryptophan (KYN/TRP) ratio in drug-free children with ADHD as compared with healthy controls. 11

Blood concentrations of tryptophan and its catabolites in people with ADHD (overall analyses)

Figure S1. Blood tryptophan (TRP) concentrations in people with ADHD as compared with healthy controls [26,27,40–43].

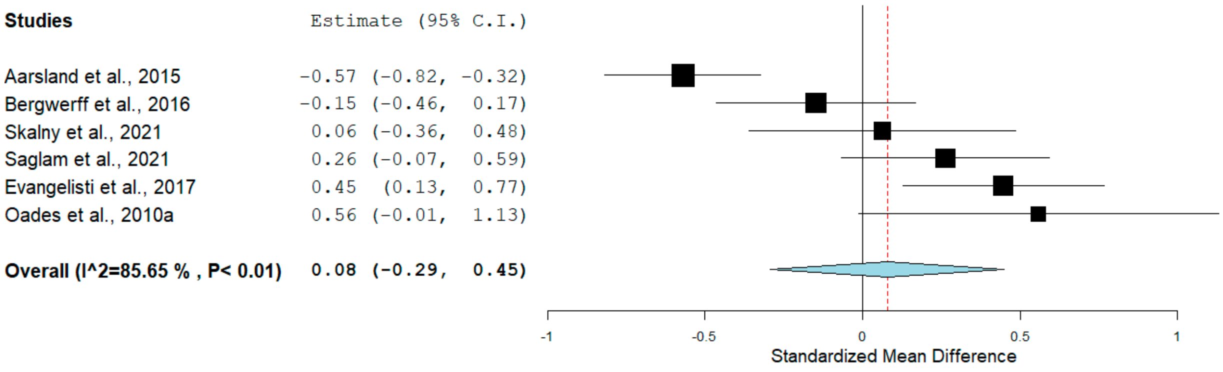

Figure S2. Serum kynurenine (KYN) concentrations in people with ADHD as compared with healthy controls [26,27,38,40,43].

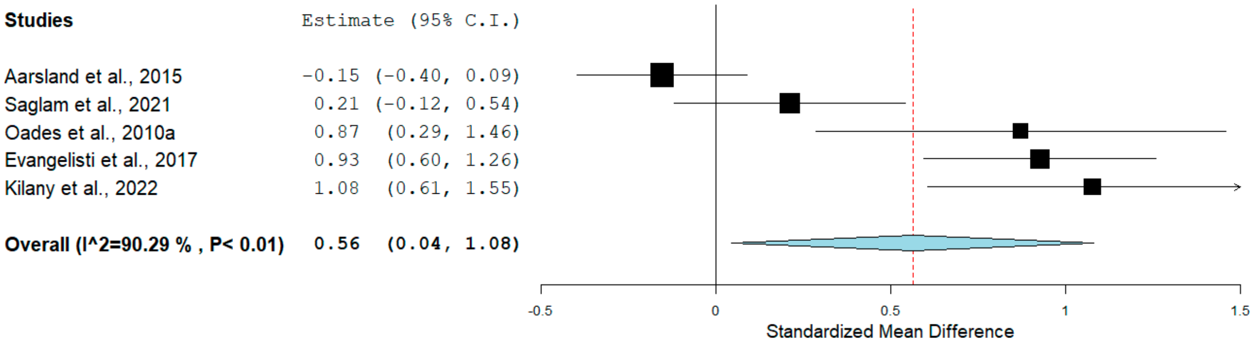

Figure S3. Serum kynurenic acid (KYNA) concentrations in people with ADHD as compared with healthy controls [26,27,40,43].

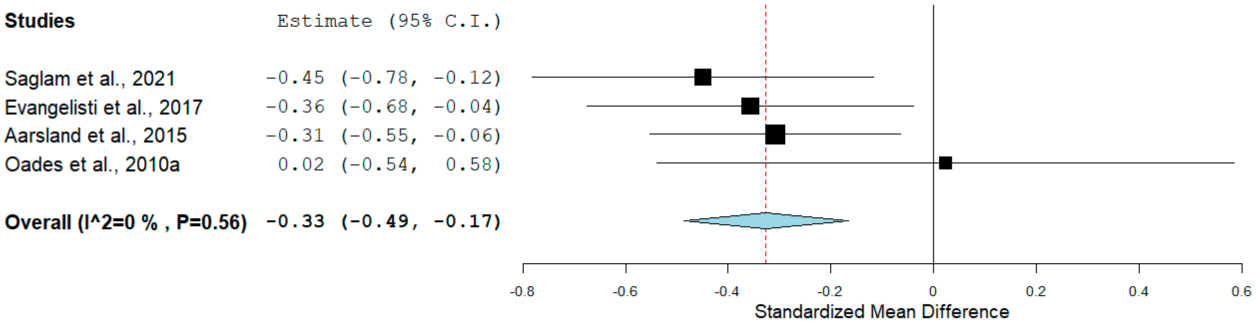

Figure S4. Serum 3-hydroxykynurenine concentrations in people with ADHD as compared with healthy controls [27,40,43].

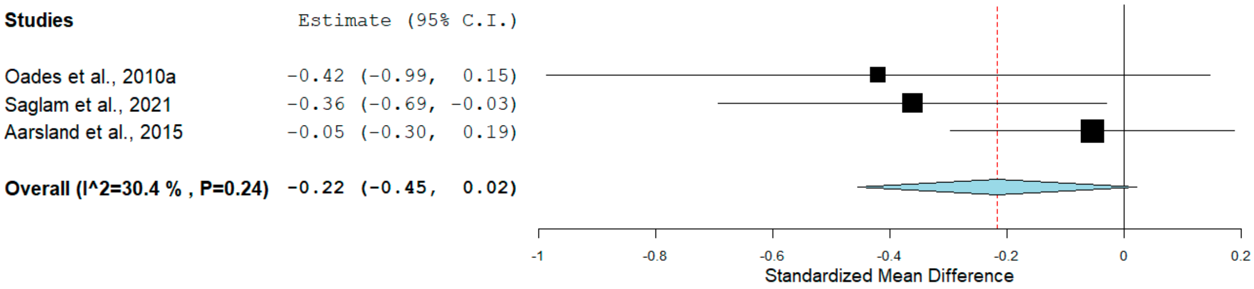

Figure S5. Serum anthranilic acid (AA) concentrations in people with ADHD as compared with healthy controls [26,27,39].

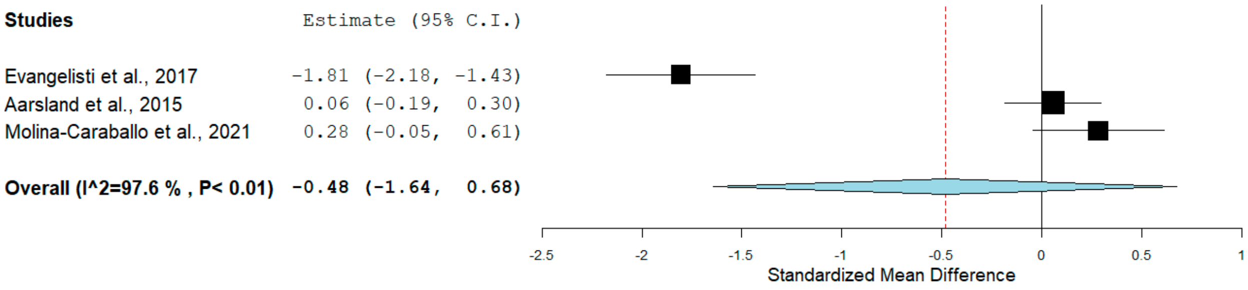

Figure S6. Serum 3-hydroxyanthranilic acid (3HAA) concentrations in people with ADHD as compared with healthy controls [26,27,40].

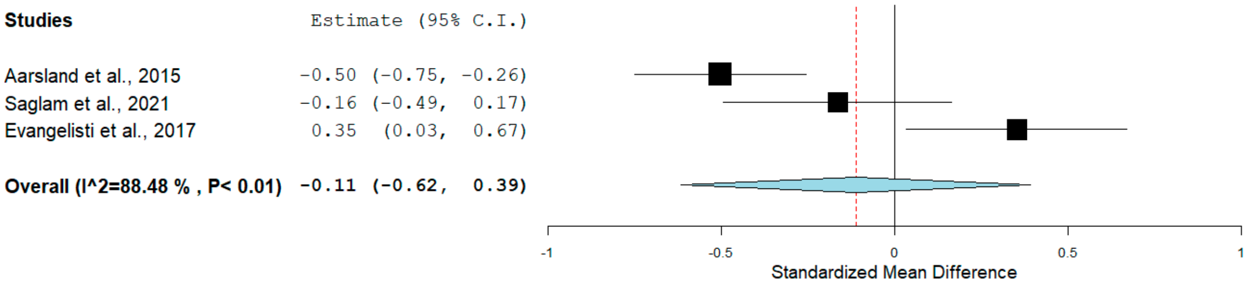

Figure S7. Serum kynurenine/tryptophan (KYN/TRP) ratio in people with ADHD as compared with healthy controls [26,27,40,43].

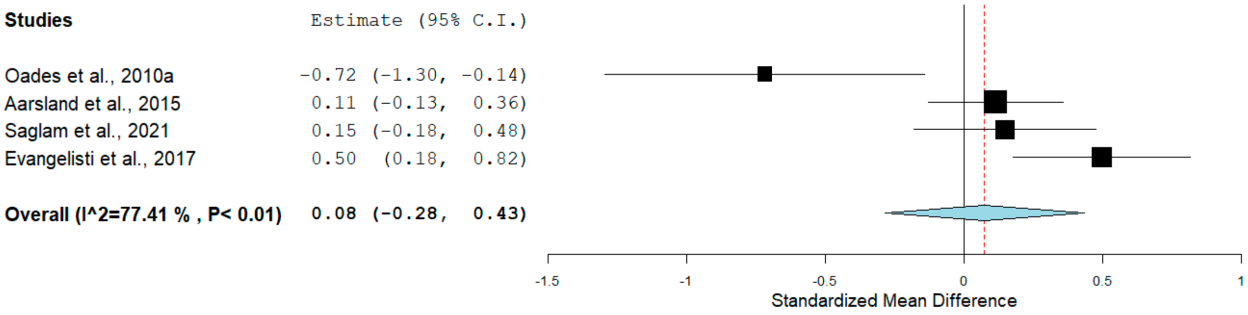

Blood concentrations of tryptophan and its catabolites in drug-free children with ADHD

Figure S8. Blood tryptophan (TRP) concentrations in drug-free children with ADHD as compared with healthy controls [26,40,41,43].

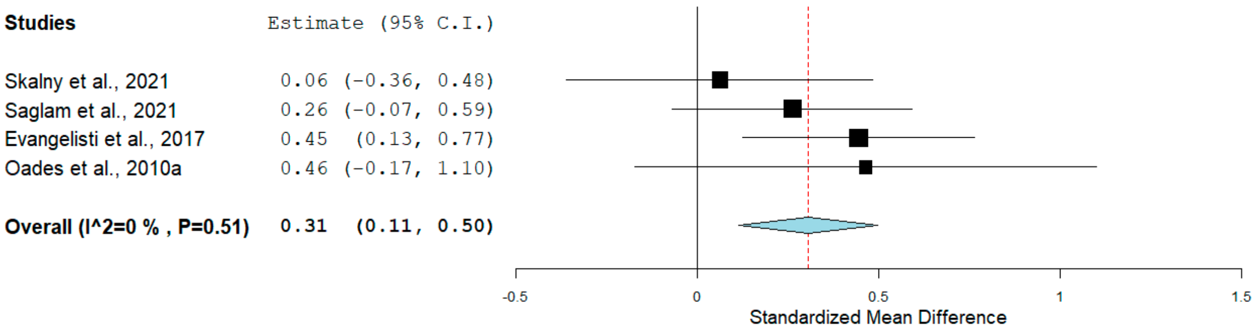

Figure S9. Serum kynurenine (KYN) concentrations in drug-free children with ADHD as compared with healthy controls [26,38,40,43].

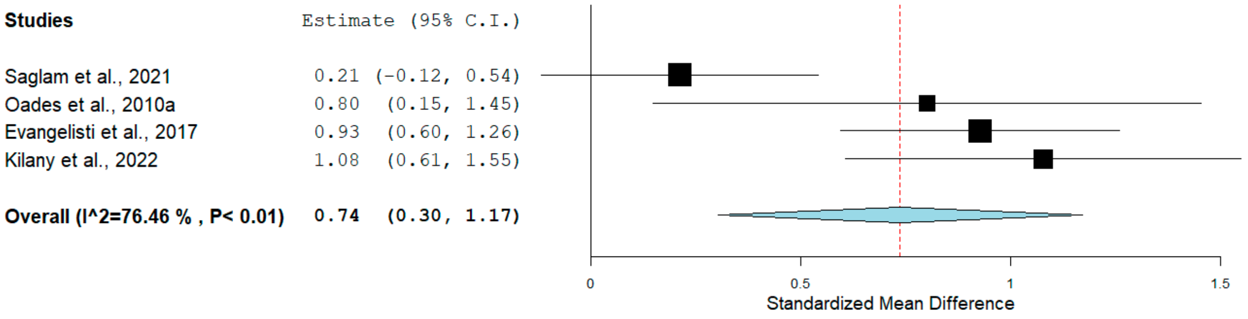

Figure S10. Serum kynurenic acid (KYNA) concentrations in drug-free children with ADHD as compared with healthy controls [26,40,43].

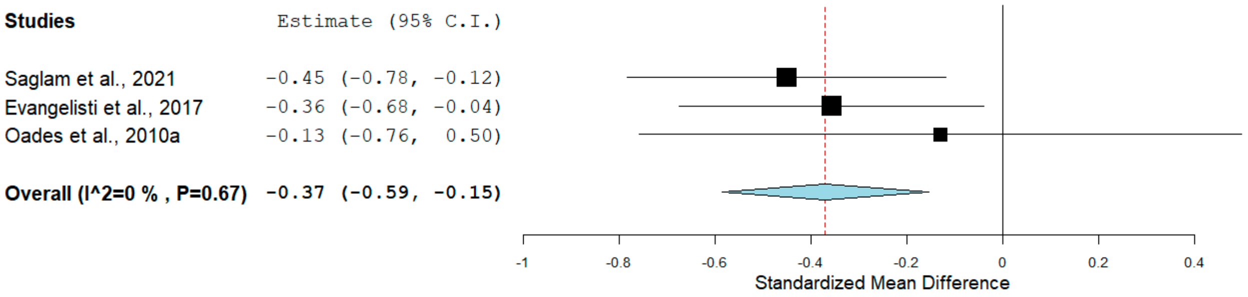

Figure S11. Serum kynurenine/tryptophan (KYN/TRP) ratio in drug-free children with ADHD as compared with healthy controls [26,40,43].

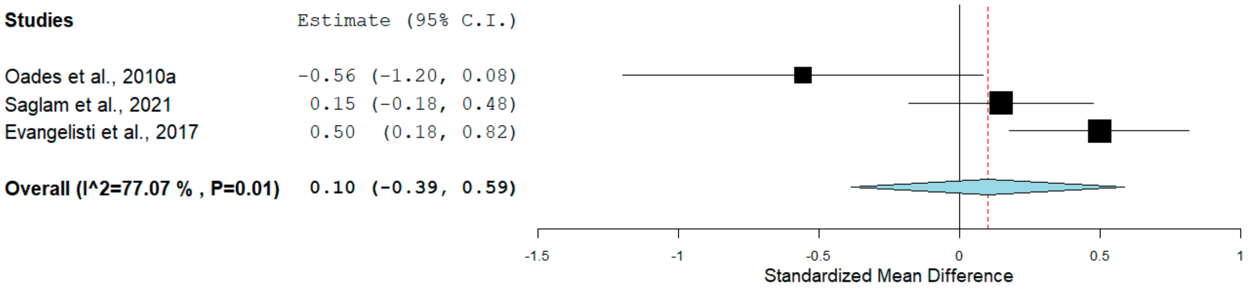

Supplement: Supplementary file 1 [file jcm-13-00583-s001.zip › jcm-2794346-supplementary.pdf]
